# Identification of Adverse Drug Events from Free Text Electronic Patient Records and Information in a Large Mental Health Case Register

**DOI:** 10.1371/journal.pone.0134208

**Published:** 2015-08-14

**Authors:** Ehtesham Iqbal, Robbie Mallah, Richard George Jackson, Michael Ball, Zina M. Ibrahim, Matthew Broadbent, Olubanke Dzahini, Robert Stewart, Caroline Johnston, Richard J. B. Dobson

**Affiliations:** 1 MRC Social, Genetic & Developmental Psychiatry Centre (SGDP), King’s College London, London, United Kingdom; 2 Pharmacy Department, South London and Maudsley NHS Foundation Trust, London, United Kingdom; 3 Department of Health Service & Population Research, Institute of Psychiatry, King’s College London, London, United Kingdom; 4 NIHR Biomedical Research Centre for Mental Health, South London and Maudsley NHS Foundation, London, United Kingdom; 5 Biomedical Research Unit for Dementia, South London and Maudsley NHS Foundation, London, United Kingdom; Hellas, GREECE

## Abstract

**Objectives:**

Electronic healthcare records (EHRs) are a rich source of information, with huge potential for secondary research use. The aim of this study was to develop an application to identify instances of Adverse Drug Events (ADEs) from free text psychiatric EHRs.

**Methods:**

We used the GATE Natural Language Processing (NLP) software to mine instances of ADEs from free text content within the Clinical Record Interactive Search (CRIS) system, a de-identified psychiatric case register developed at the South London and Maudsley NHS Foundation Trust, UK. The tool was built around a set of four movement disorders (extrapyramidal side effects [EPSEs]) related to antipsychotic therapy and rules were then generalised such that the tool could be applied to additional ADEs. We report the frequencies of recorded EPSEs in patients diagnosed with a Severe Mental Illness (SMI) and then report performance in identifying eight other unrelated ADEs.

**Results:**

The tool identified EPSEs with >0.85 precision and >0.86 recall during testing. Akathisia was found to be the most prevalent EPSE overall and occurred in the Asian ethnic group with a frequency of 8.13%. The tool performed well when applied to most of the non-EPSEs but least well when applied to rare conditions such as myocarditis, a condition that appears frequently in the text as a side effect warning to patients.

**Conclusions:**

The developed tool allows us to accurately identify instances of a potential ADE from psychiatric EHRs. As such, we were able to study the prevalence of ADEs within subgroups of patients stratified by SMI diagnosis, gender, age and ethnicity. In addition we demonstrated the generalisability of the application to other ADE types by producing a high precision rate on a non-EPSE related set of ADE containing documents.

**Availability:**

The application can be found at http://git.brc.iop.kcl.ac.uk/rmallah/dystoniaml.

## Introduction

In the digital era many healthcare providers have transitioned from keeping paper copies of patient and prescription data to electronic records. Although the concept behind electronic health records (EHRs) was primarily to retain documentation of a patient’s medical history, it is now apparent that these digital data sets represent a valuable resource for research. However, EHRs are optimised for day-to-day clinical use, not for research, resulting in data sets that are often unstructured, ill-defined and arduous to analyse at scale. Despite these challenges, a number of studies have made use of the rich data in EHRs to mine details relating to adverse drug events (ADEs) for example [1].

Adverse drug reactions (ADRs; ADEs where drug causality is established) are troublesome and potentially fatal outcomes of medication treatment and result in extra expense for health care providers. The ability to mine for, and eventually predict, occurrences of ADRs could have significant patient and cost benefits in the future [2]. A 2004 analysis of 18,820 patients showed that the projected annual costs for ADRs that led to hospital admissions would total 5466m [3]. In addition, a US study reported that there were 2341 ADR related deaths from data collected between 1999 and 2006. Annual mortality rates ranged from 0.08 to 0.12 per 100,000, increasing significantly over time at a rate of 0.0058 per year [4]. After the initial testing phase of a drug, spontaneous reporting systems, such as the UK Yellow Card Scheme, are the primary means for identifying suspected ADRs. These systems are reliant on patient and clinician data entry and many ADRs are under reported [5].

### ADE Knowledge Discovery in Electronic Health Records

A number of studies have used text-mining techniques and natural language processing (NLP) tools in EHRs to identify ADEs and establish their causal relationships with drugs. Initially, to detect adverse events from clinical text, simple string matching approaches were applied.

Honigman et al (2001) [6] used notes from the outpatient department of Brigham and Women’s Hospital (Boston, USA) to computationally identify ADEs. String matching was used to identify Micromedex M^2^D_2_ [7] medical data dictionary concepts in: ICD 9 diagnosis codes; patient drug allergy data; computer event monitoring (laboratory tests, prescription data) and free text clinical notes. Possible ADEs were subsequently manually reviewed. The study identified 864 possible ADEs. In a similar approach, Murff et al (2003) [8] investigated adverse events resulting from medical management records rather than patient’s underlying conditions. A computer-based string matching tool was applied to search free-text discharge summaries for trigger words, consisting of a broad range of adverse events. A manual review of the discharge summaries showed 44.8% (327 of 730) of the search term hits were true adverse events representing 131 ADEs. Field et al (2004) [9] conducted a study on patients aged over 65 to detect possible drug-related incidents and identified 1,523 ADEs during a one-year period, of which 421 (28%) were deemed preventable.

In each of these studies, the cohort sizes were limited because the approach required manual review of all results. More recently, studies have taken advantage of NLP tools that have come to replace simple string matching as a major method for detecting adverse events from clinical free text. The cohort sizes of subsequent studies increased, and some studies even applied NLP tools on the whole set of EHR. One such study was performed by Hazelhurst et al (2009) [10], whereby the researchers conducted a study of outpatients to identify vaccine related gastrointestinal adverse events. They used MediClass [11] (an automated classification system) and programmed it to identify vaccine related clinical concepts and linguistic structures used in clinical notes to extract vaccine related adverse events. After encoding the knowledge into MediClass, it detected 319 possible adverse events out of which 181 were true positives (determined upon manual review). However there were some limitations with the study. The manual review was conducted by the author rather than independent coders and the ICD 9 codes do not have good coverage for vaccine related ADEs.

Wang et al (2009) [12] conducted a study using notes from the inpatient department of Presbyterian Hospital, New York. They applied a modified version of the MedLEE NLP tool [13] and used MedDRA symptoms to detect adverse events from discharge summaries. The recall and precision were 75% and 31% and the application detected 132 ADE related to the seven medications. They went on to conduct another study [14] on the same EHR data source and ran MedLEE by applying filters (information extraction modules) to capture symptoms and adverse events caused by using medication during the course of hospitalisation. They applied regular and contextual filters in order to reduce the amount of confusing information. In the regular filter they avoided family history (mother suffered from ADE), past events (patient suffered from ADE last year) and negation (patients shows no signs of ADE). In the contextual filter they kept the clinical information where it was indicated that the drug was administered prior to the adverse event (i.e. establishing the correct time sequencing). Assessment showed that applying the filters improved recall (In Symptoms: from 0.85 to 0.90; ADE: from 0.43 to 0.75) and precision (In Symptoms: from 0.82 to 0.92; ADE: from 0.16 to 0.31).

In another study using the inpatient notes from the department of Presbyterian Hospital, New York, Haerian et al (2010) [15] used the MedLEE natural language processor with a filter that was built with expert knowledge on discharge summaries for patient with elevated creatine kinase serum. They investigated the ADE Rhabdomyolysis resulting from myopathy inducing medication and successfully identified 165 ADE with 96.7% correctly identified rate.

Finally, Eriksson et al. (2013) [16] described methods to develop an adverse event dictionary in Danish clinical narratives. They used Python libraries for NLTK and identified 35,477 unique possible ADEs in a Danish psychiatric hospital’s EHR. Manual inspection was performed to validate the ADEs, resulting in precision of 89% and recall of 75%.

The aim of the study described here was to develop a generic natural language processing (NLP) tool for identifying adverse drug events (ADEs) from text fields in English-language mental healthcare records. We define an ADE as any event that could be an ADR; however, at this stage we did not attempt to establish causality from the record (e.g. relating to the agent potentially responsible) but instead simply sought to ascertain the symptom/event itself. The tool was initially built to identify the four key extrapyramidal side effects (EPSEs) associated with antipsychotic treatment: dystonia, Parkinsonism, akathisia, and tardive dyskinesia. EPSEs are a group of movement disorders ranging from sustained contractions of the muscle, twisting or repetitive movements or abnormal postures in dystonia [17], an inner sensation of restlessness resulting in a patient being unable to remain motionless with akathisia. Parkinsonism, also called Parkinsonresulting in a patient being unable to remain motionless with akathisiaDisplayText><record><rec-number>124</rec-number><foreign-keys><key app = "[18]. Tardive dyskinesia, associated with long-term antipsychotic use, manifests as slow repetitive movements [19]. Although rarely life threatening, EPSEs can be debilitating leading to social anxiety and embarrassment, as well as potentially causing non-adherence to medication regimes and risking relapse [20]. In addition, prophylaxis and treatment of EPSEs usually requires further pharmacotherapy and the potential for additional ADRs. Understanding them further and being able to assess the potential for exposure within specific groups is therefore an important challenge. In a second step, the tool was applied to an unrelated mix of rare and common ADRs, described within the Medical Dictionary for Regulatory Activities (MedDRA), an international medical terminology dictionary in wide clinical use. The performance in these ‘unseen’ ADEs was assessed for generalizability of the approach.

## Methods

### Data Source

The development of NLP software to detect ADEs was carried out in a large mental healthcare EHR data resource. The South London and Maudsley NHS Foundation Trust (SLaM) is the largest mental health provider in Europe serving a population of over 1.2 million residents from four London boroughs (Croydon, Lambeth, Lewisham and Southwark) [21]. The SLaM EHR, the Electronic Patient Journey System (EPJS), is typical of many such systems in that it stores much of its clinical records and prescribing information in an unstructured free text format. All use of data in our study is covered by a pre-existing ethical approval covering data analysis (Oxford C Research Ethics Committee, reference 08/H0606/71+5; renewed on 4.7.2013 for a further 5 years).

As of October 2012 there were over 200,000 patient records held in EPJS comprising over 20,000,000 free text documents including correspondence, discharge letters and events, increasing at a rate of 300,000 new documents per month. In order to create a resource for research, the Clinical Record Interactive Search System (CRIS) [22], a de-identified version of the EHR, was developed in 2007 and further enhanced with language processing tools to extract information from the vast amount of free text format data stored within this database.

### Identification of Extrapyramidal Side Effects

**Fig 1:**
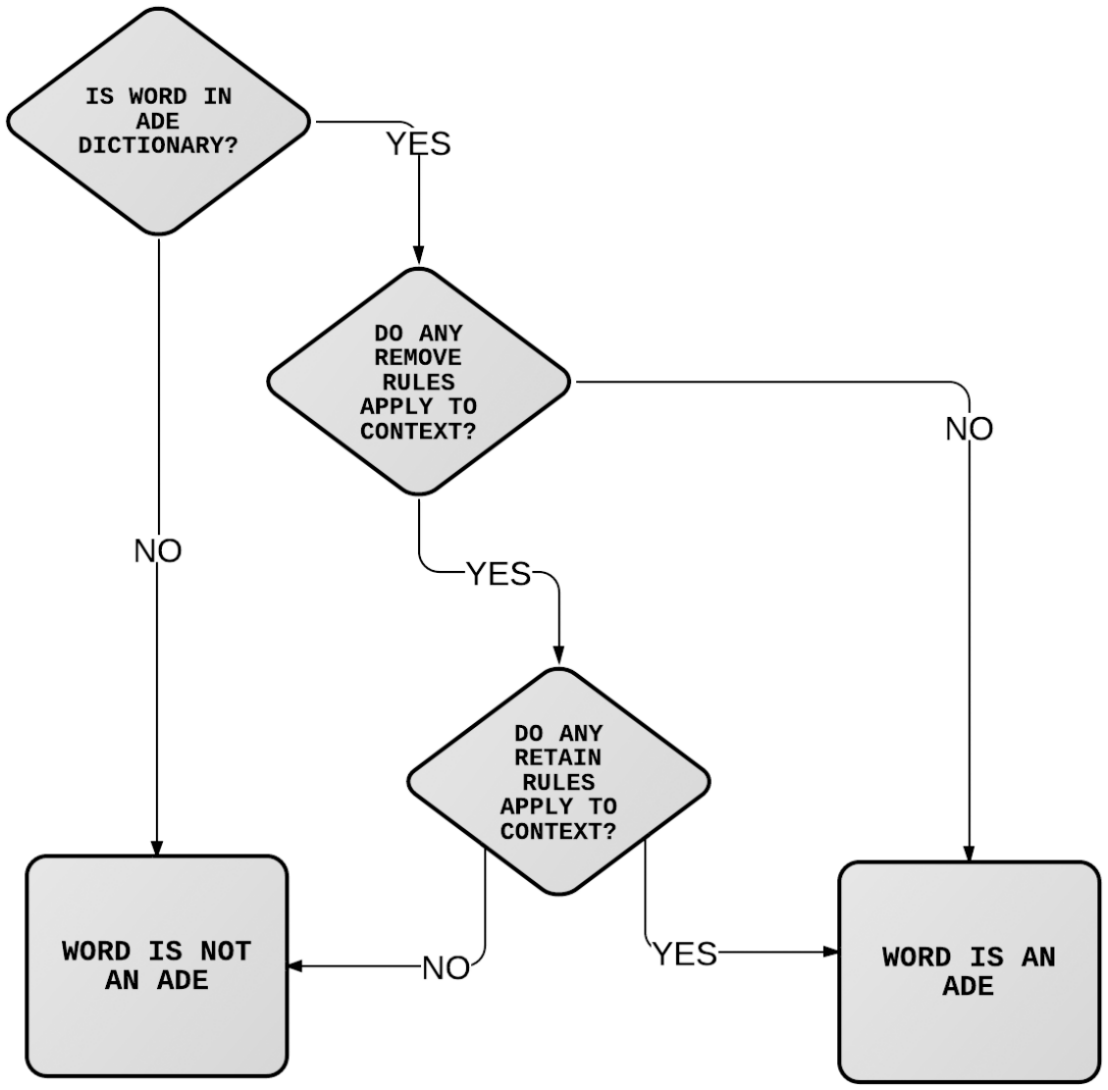
Remove and Retain rules. Flow diagram representing the use of the Remove and Retain rules to identify ADE instances.

We used the GATE (General Architecture for Text Engineering) NLP framework [23, 24] to develop an application to extract ADE information from free text fields over the whole of CRIS regardless of diagnosis. We trained the ADE tool on detecting EPSEs during its development. First, we defined a dictionary of EPSE ADE terms, including synonyms and alternate spellings. The application initially identifies all mentions of these terms as potential ADEs and then applies a series of rules to remove terms used in a different context. Removal rules can be overridden by ‘retain rules’ in cases where there is additional clear evidence that the word describes a real ADE. The process is illustrated in the flowchart in Fig 1.

Rules were defined using the Java Annotation Patterns Engine (JAPE) within GATE. Removal rules were written to handle cases where ADE terms were negated; in instances where clinicians were warning about, or monitoring for potential ADEs; names of charities or research organisations for ADEs; mentions of ADEs referring to a subject other than the patient and cases where mentions indicated uncertainty in diagnosis. It was more important to ensure that identified ADEs were real than to ensure that all ADE mentions were identified, so these rules were developed favouring precision over recall. As ADEs are often mentioned multiple times in a patient record, a missed ADE in one document can be expected to be identified in another document, meaning recall may actually be higher than reported by the tool. To further improve recall we also defined a number of retain rules that could override removal rules when the context made it clear that ADE was present in the patient. Specifically we retained cases where the definite article or a possessive pronoun immediately preceded an ADE: e.g. ‘The patient does not think the dystonia was painful’. We also defined a dictionary of commonly used diagnostic phrases that constitute strong evidence of a real ADE: e.g. be expect’, We also defin’. Table 1 shows examples of text where Removal and Retain rules were required.

**Table 1:** Examples of rule firing annotations. Rules were deployed within the JAPE of GATE.

Text where Removal rules are used	Text where Retain rules are used
‘ext where ***not*** have dystoniat	‘ave dystoniatain ***her*** dystonia had become worsee
‘dyston***denied*** any dystonic reactionsr	‘any dystonic re***his*** dystonia had become a probleme
‘dystonia had bec***possibility*** of dystonia with ZZZZZp	‘of dystonia w***her*** dystonia was severeZ
‘dystonia was ***Society*** have always been a useful resource for patientse	‘have always been a useful resou***his*** dystonia being reducedf
‘***If*** Dystonia develops give procyclidine dosec	
‘***Check for*** any dystonic reactione	
‘any dyst***mother*** had developed dystonia many years agoo	

**Fig 2:**
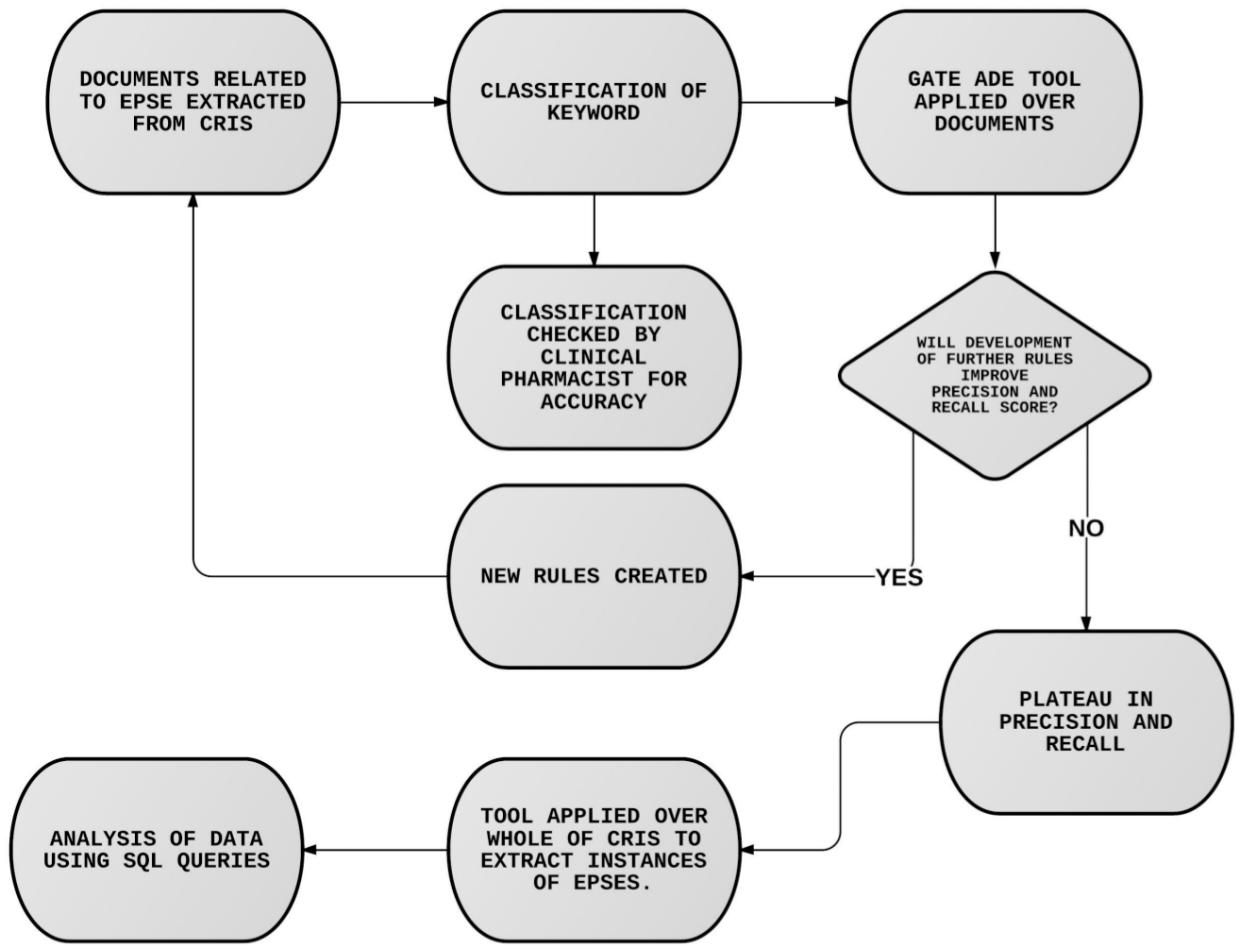
Iterative ADE tool development process. Flow diagram showing the iterative approach taken in development of the tool.

**Fig 3:**
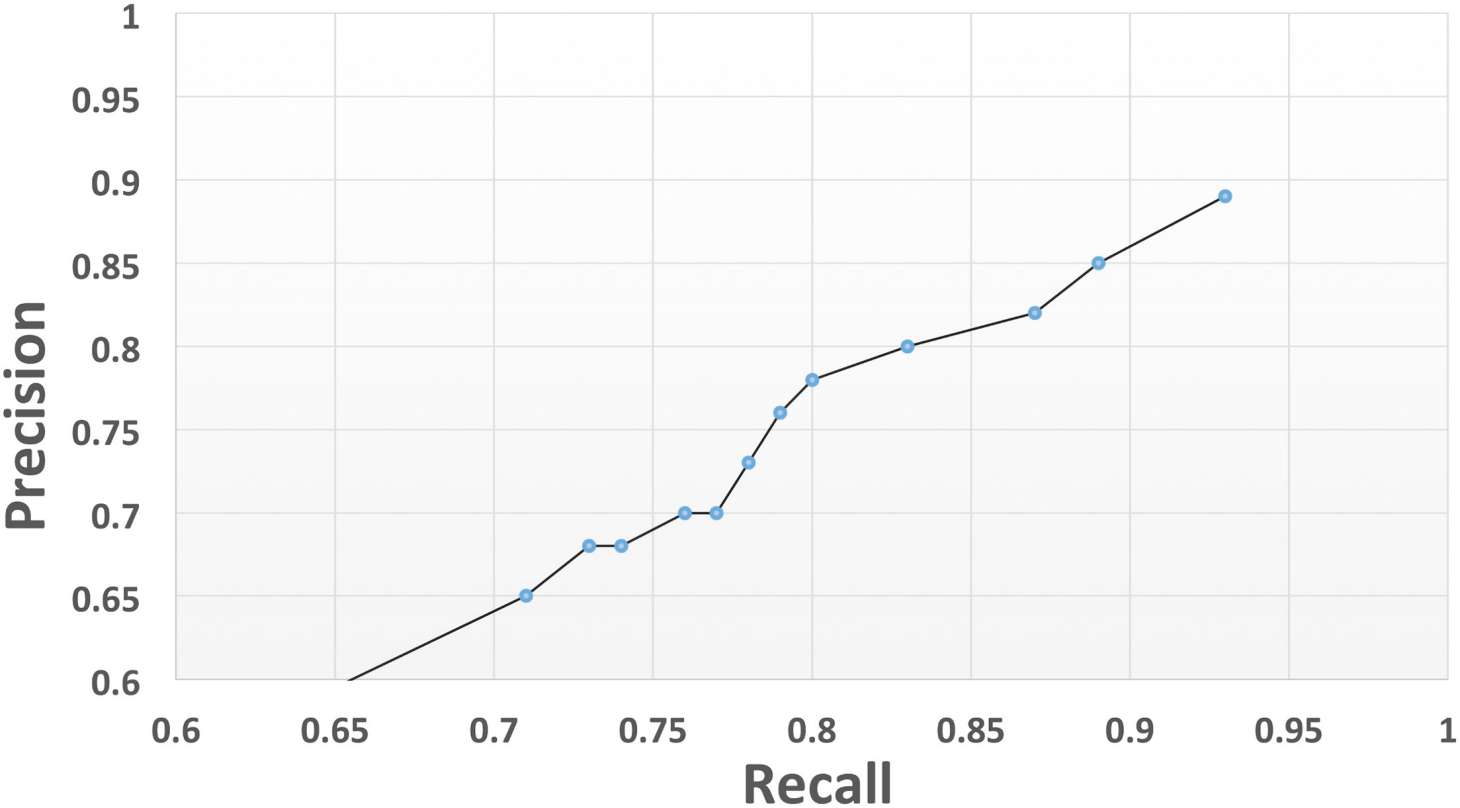
Precision and recall plot for dystonia JAPE rule development. The plot shows the evolution of the performance over the iterative JAPE rule development process for dystonia.

The development phase of the application started with the identification of dystonia ADEs and followed an iterative path whereby rules were developed, the application performance was tested and misclassifications were used to create new and improved rules. (See Fig 2). A different set of manually annotated documents were used for each round of testing. Table 2 shows JAPE rules that were implemented for the dystonia application and corresponding improvement in precision and recall, also shown in Fig 3. Once a plateau had been reached for dystonia, development continued for akathisia, Parkinsonism and tardive dyskinesia EPSEs.

**Table 2:** Corresponding precision and recall by applying each set of JAPE rules on dystonia corpus.

Stage	JAPE Rule	Purpose of rule	Precision	Recall
	Keyword Search only	Baseline estimates. Not used in iteration process.	62%	98%
1	Negation Before ADE terms	This rule negates the ADE terms which appear before the negation terms. The terms are present in the ‘negationbefore’ gazetteer.	71%	65%
2	Negation After ADE terms	This rule negates the ADE terms which appear after the negation terms. The terms are present in the ‘negationafter’ gazetteer.	73%	68%
3	Organisation	This rule negates the ADE terms appear before or after organisation terms (i.e. dystonia workshop). The terms are present in the ‘organisation’ gazetteer.	74%	68%
4	Symbols and punctuations	These rules negate the ADE terms if symbols and punctuations (like; ‘?’, ‘/’ and ‘@’) appear before or after the ADE terms, with or without any space tokens.	76%	70%
5	Other Person	This rule negates the of ADE terms if other person is discussed in the sentence. The terms are present in the ‘people’ gazetteer.	77%	70%
6	Monitor	These 2 rules negate the ADE terms if clinicians are monitoring for ADE. The terms are present in the ‘monitor’ gazetteer.	78%	73%
7	Negative effects	These 7 rules negate the ADE terms where clinicians are explaining, informing, potential and common ADE to the patient. The negative effects terms are present in ‘negseffect’ gazetteer.	79%	76%
8	Single words around ADE	These 2 rules negates the ADE terms where words appears before the ADE (i.e. ‘like’, ‘rates’) and after the ADE (i.e. ‘consider’)	80%	78%
9	Diagnosis	This rule negate the ADE terms which are actually the diagnosis (i.e. ego dystonia) The terms are present in the ‘diagnost’ gazetteer.	83%	80%
10	Drugs Effects / Vaccine	These 6 rules negate the ADE terms where clinicians are discussing/explaining the side effects of a drug. The side effects terms are present in the ‘druglink’ gazetteer.	87%	82%
11	If statement	These 2 rules negate the ADE terms which are hypothetically discussed as a reaction/indication. The ADE indication terms are present in ‘ADRin’ gazetteer.	89%	85%
12	Retain Rule	These 3 rules un-negate the negation if ADEs terms are present within close proximity of patient name and ADE indication terms present in ‘ADRin’ gazetteer.	93%	89%

Performance was assessed using a quality assurance (QA) tool built into the GATE software. Batches of 200 documents with the mention of the specific ADE contained within them (e.g. dystonia) were extracted from the CRIS database as XML files. The Text Hunter tool, developed in-house and available online, (http://sourceforge.net/projects/texthunter) was used to enable a clinical pharmacy technician to assign a positive or negative classification to each ADE mention. ADE mentions were classified as positive, even when they were clearly indicating a past event. Performance was assessed by considering precision and recall of the application compared to manual annotation. Once the development process was complete, the application was run over all free text fields in CRIS on a high performance computer cluster hosted behind the SLaM NHS firewall, storing the results in a Microsoft SQL server instance for downstream analysis.

### Prevalence of Extrapyramidal Side Effects in patients with serious mental illness

To demonstrate the utility of the approach, we investigated the frequency of EPSEs within the 17,995 patients represented on CRIS who had received a diagnosis of a serious mental illness (SMI; schizophrenia, bipolar disorder, schizoaffective disorder) [25] between 2007–2013 which included alive and deceased patients,. We removed deceased patients from this cohort (n = 2087). The diagnosis of each patient was selected as the most recent diagnosis recorded prior to 1st of January 2014. Diagnosis was assigned from a mandatory structured field in the clinical record, which records this information using ICD-10 categories and/or an in-house GATE application that mines text strings associated with diagnosis statements in clinical correspondence [26]. Mortality (an exclusion criterion) was ascertained through routine tracing of past and current cases on EPJS against the national register [27]. The prevalence of EPSEs across groups split by age (on 1st of January 2014), ethnic group, gender and SMI diagnosis subsets were tested using chi-squared tests.

### Generic capability of the tool to identify adverse drug events

We tested the generic capability of the unmodified retain and remove rules developed within the tool by applying it to a range of ADEs unrelated to EPSEs but of interest in relation to the treatment of schizophrenia and bipolar disorder: alopecia, convulsions (seizures), hypersalivation, myocarditis, nausea, pneumonia, Stevens-Johnson syndrome, and tachycardia, chosen to represent a range from rare to common and mild to severe.

### Annotator agreement

To explore and quantify reliability, a second manual annotator, a clinical pharmacist, independently classified the ADE mentions within a test corpora of documents for two EPSE ADEs, namely akathisia and dystonia, and two non-EPSE ADEs, alopecia and myocarditis. We rated the level of agreement between the two classifiers with a percentage score and Cohendystonia.

## Results

### Identification of Extrapyramidal Side Effects

Performance metrics for the NLP applications in test corpora, following the iterative model building step, are displayed in Table 3. The application of the JAPE rules substantially improved precision over a keyword search term alone. Recall statistics were also maintained at satisfactory levels in most instances. The tool performed least well on the Parkinsonism EPSE, reaching a plateau of 0.85 precision and 0.88 recall. The other EPSEs returned precision scores of >0.90 and recall >0.86.

**Table 3:** Performance metrics for JAPE rules identifying extrapyramidal side-effects (EPSEs).

EPSE	Precision		Recall	
	Using keyword search only	With Remove and Retain rules applied	Using keyword search only	With Remove and Retain rules applied
Dystonia	0.62	0.93	0.98	0.89
Akathisia	0.61	0.92	>0.99	0.86
Parkinsonism	0.58	0.85	0.94	0.88
Tardive dyskinesia	0.89	0.97	>0.99	0.90

### Prevalence of Extrapyramidal Side Effects in patients with serious mental illness

Descriptive data on EPSE prevalence in patients with an SMI diagnosis are summarised in Table 4. Akathisia was the most frequently recorded EPSE in all groups. Significant heterogeneity was found for most comparisons although patterns of associations differed between the EPSEs. Akathisia showed no significant differences across the age groups. Dystonia was more commonly identified in younger compared to older patients, whereas the opposite was the case for Parkinsonism and tardive dyskinesia which appeared to be more prevalent in the older age groups with low incidence in the young. Men had higher incidence of recorded dystonia and akathisia than women but there were no gender differences in Parkinsonism or tardive dyskinesia. Considering ethnicity, akathisia and Parkinsonism were most frequently recorded in Asian groups and dystonia and tardive dyskinesia most prevalent in black groups. In terms of diagnosis, all EPSEs were lowest in bipolar patients and highest in schizoaffective disorder patients.

**Table 4:** Recorded EPSE frequencies for patients with serious mental illness (SMI) according to demographic status and diagnosis. The numbers reflect a cohort of 12879 patients from 2007 to 2013.

EPSE		Dystonia	Akathisia	Parkinsonism	Tardive Dyskinesia
Total Number of Patients		390	750	440	324
**Age Group**	**Cohort Size**	**EPSE Prevalence**			
Under 21	318	5.97%	8.18%	3.46%	0.63%
21 to 30	2106	4.51%	6.03%	2.71%	1.47%
31 to 40	3018	3.61%	5.40%	2.78%	1.46%
41 to 50	3249	2.65%	6.25%	2.22%	2.28%
51 to 60	2119	2.27%	5.85%	3.21%	2.41%
61 to 70	1129	1.86%	5.93%	6.20%	5.23%
71 to 80	677	1.33%	4.73%	9.31%	7.39%
Above 80	263	1.14%	3.04%	5.70%	4.94%
Chi-Square value (7 df) P-Value		49.568 <0.001	10.648 0.155	123.193 <0.001	10.648 <0.001
**Gender**	**Cohort Size**	**EPSE Prevalence**			
Male	6969	3.49%	6.50%	3.26%	2.55%
Female	5910	2.49%	5.03%	3.60%	2.47%
Chi-Square value (1 df) P-Value		10.881 <0.001	12.684 <0.001	1.165 0.280	0.092 0.762
**Ethnicity**	**Cohort Size**	**EPSE Prevalence**			
White	5788	2.32%	6.10%	3.27%	2.16%
Black	4682	4.44%	5.55%	3.55%	3.25%
Asians	861	2.32%	8.13%	6.04%	3.14%
Other	1548	1.81%	4.33%	2.13%	1.29%
Chi-Square value (3 df) P-Value		51.214 <0.001	16.088 <0.001	26.332 <0.001	23.990 <0.001
**SMI**	**Cohort Size**	**EPSE Prevalence**			
Schizophreniform	8411	3.11%	5.91%	3.40%	2.87%
Bipolar	3208	2.03%	3.99%	2.77%	1.00%
Schizoaffective	1260	5.00%	9.92%	5.16%	4.05%
Chi-Square value (2 df) P-Value		27.867 <0.001	58.342 <0.001	15.607 <0.001	46.399 <0.001

### Generic capability of the tool to identify adverse drug events

Table 5 displays performance metrics for the NLP tool applied to non-EPSE ADEs. In summary, the tool performed well over most ADEs, but least well for myocarditis and Stevens-Johnson syndrome. Precision increased with the application of Remove and Retain rules compared to a keyword search.

**Table 5:** Performance metrics for JAPE rules identifying selected other (non-EPSE) adverse drug events (ADEs).

ADE	Precision		Recall	
	Using keyword search only	With Remove and Retain rules	Using keyword search only	With Remove and Retain rules
Alopecia	0.87	0.92	>0.99	0.76
Convulsions (seizures)	0.68	0.88	>0.99	0.81
Hypersalivation (sialorrhea)	0.91	0.95	>0.99	0.82
Myocarditis	0.34	0.64	>0.99	0.69
Nausea	0.68	0.96	0.96	0.74
Pneumonia	0.77	0.84	>0.99	0.82
Stevens-Johnson syndrome	0.29	0.59	>0.99	0.73
Tachycardia	0.85	0.93	>0.99	0.83

### Annotator agreement

Inter-annotator agreement statistics are summarised in Table 6 and range from 88% (Cohenble ement statistics are summarised in Table-EPSE AD.

**Table 6:** Inter-annotator agreement test results.

ADE	Agreement (%)	Cohenment (%)ator a
Akathisia	88%	0.74
Alopecia	92%	0.70
Dystonia	96%	0.91
Myocarditis	88%	0.69
Parkinsonian	90%	0.80

## Discussion

We describe the development of an NLP tool to identify ADEs within free text fields, such as case note entries and correspondence, from a large mental health EHR-derived database. Text processing rules were initially constructed to identify EPSEs and the distributions of these were described in a sample of patients with serious mental illness. The tool was trained initially on the four principal EPSEs associated with antipsychotic pharmacotherapy, but was developed with the aim of producing a generic set of rules that would be capable of identifying ADEs beyond EPSEs. With this in mind it was important not to over-train the application for EPSE identification specifically. As a result, we found the rules performed well in identifying a range of other ADEs.

A number of challenges were encountered in the development of the application. For example, of the EPSEs targeted, the tool performed least well in identifying Parkinsonism. This was probably because of a higher risk of false positive annotations due to Parkinsonism being mentioned in contexts unrelated to ADE instances (for example, because of Parkinson’s disease itself). In general, many instances of potential ADEs were found to be ambiguous, potentially because of diagnostic uncertainty and/or clinical reluctance to record an ADE as definitive. Because of the priority we placed on precision over recall, where there was any doubt around an ADE diagnosis the instance was classified as negative. For this stage of development, the NLP application was designed simply to identify text indicative of a given ADE regardless of timing. Some of the recorded ADEs observed during the manual annotation process related to past instances and this should be considered when interpreting findings. Further development of the application is ongoing to enable future studies dependent on temporal relationships; for example, those investigating timing in relation to medication use.

Obtaining a good recall score on an ADE was reliant on a broad keyword selection within the gazetteer, incorporating as many descriptions of the ADE as possible. For example, there were a number of alternative spellings of akathisia in the source records (e.g. acasthisia, acathisia, akithisia) which required consideration when developing the gazetteer, and which will need further consideration when applying the tool over the wider MedDRA list of ADEs.

Lower precision and recall statistics were found for the more rare but serious ADEs. This tended to occur because they were more frequently cited in text fields as a warning rather than an occurrence. For example, myocarditis is a rare side effect of clozapine medication [28], but due to its severity, it was often mentioned as a potential consideration or as a recorded warning. These instances were classified as negative in the annotation process but it proved more challenging to produce Remove rules that would identify each one of these false positives.

Despite their importance in mental healthcare and psychopharmacology, EPSEs have been relatively understudied in naturalistic environments [29, 30]. As such this analysis demonstrates the power of secondary use of clinical records for research. However, these data have a number of caveats. Most importantly, the data reflect ADEs that are both recognized and recorded and thus are likely to underestimate the true situation, further reduced by the design of the NLP application to focus on unambiguous instances and ignore tentative terminology. EPSE recognition is also considered to be challenging at a clinical level: for example, the misdiagnosis of akathisia as agitation [31] or dystonia and akathisia as features of the underlying mental disorder [32]. Additionally, a study by Somers et al 2003 reported that spontaneous reporting by physicians and nurses on a geriatric ward revealed considerably fewer ADRs than a patient interview by a pharmacist [33]. However, in the absence of any other means of routine recording of these ADEs, our approach at least allows some scope for surveillance and targeted intervention.

Dystonia was more frequently recorded in the young and in males and reduced linearly with age, supporting previous findings [34]. Akathisia remained relatively consistent in recorded rates through the ages. We were unable to find any previous studies supporting age in being a significant factor in the development of akathisia. Prevalence of recorded Parkinsonism and tardive dyskinesia, on the other hand, display a progressive increase with age, with Parkinsonism displaying a slight dip in the 41–50 group. This increase through the ages is understandable as tardive dyskinesia is more associated with long-term antipsychotic use and Parkinsonism is more common in elderly females [35].

Recorded EPSEs varied noticeably in prevalence between ethnic groups. In particular, akathisia and Parkinsonism were more commonly recorded in patients of an Asian ethnicity whereas dystonia and tardive dyskinesia were more commonly recorded in patients of black ethnicity. There is some evidence that prescribing in psychiatry varies between ethnic groups. While this may reflect differences in hepatic metabolism of these drugs, variations in prescribing may also relate to prejudicial clinical practice [36]. Over 50% of Asian people have intermediate metabolism of cytochrome P450 2D6 subtype (CYP2D6), one of three important enzymes metabolising antidepressants, antipsychotics and benzodiazepines. Poor metabolism of CYP2D6 leads to higher plasma levels of the drug in question with a consequently raised risk of developing EPSEs [36]. This may, in part, explain the higher recorded frequencies of akathisia and Parkinsonism within the Asian population in our cohort. Black people with a mental illness are more likely to be diagnosed with schizophrenia over non black people and have been found to be both more likely to receive a depot antipsychotic and to receive higher doses of these agents in a study based on SLaM patients in the 1990s [37]. However, more recent studies (based on the same patient population) did not find significant differences in antipsychotic type, dose or any other aspects of antipsychotic prescribing between black and white patients.[38]. Hencehe higher levels of recorded dystonia and tardive dyskinesia observed in black patients in our cohort cannot necessarily be explained by differences in antipsychotic prescribing and this point would require further investigation.

Gender was significantly associated with recorded rates of dystonia and akathisia but not Parkinsonism and tardive dyskinesia. We had expected higher recorded rates of Parkinsonism in females over males, in accordance with our literature findings. There were higher recorded rates of dystonia and akathisia in male patients over female. Male gender is a risk factor for development of dystonia and our results support this [39]. Risk factors for akathisia are not completely understood.

All EPSEs were differentially associated with SMI diagnosis, most noticeably schizophreniform and schizoaffective patients with increased rates of akathisia compared to bipolar patients. This is not surprising as antipsychotics are the most common treatment regimen for schizophreniform and schizoaffective [40], whereas bipolar disorder patients are typically treated with mood stabilisers such as lithium and valproate over antipsychotics [41].

## Conclusion

As well as providing important and novel findings on EPSEs, the NLP tool we built demonstrates utility in wider ADE extraction. In the future we will extend and evaluate the tool across ADEs listed within MedDra, to develop and introduce supplementary applications to differentiate current from past events, and to incorporate the ADE application within wider CRIS NLP developments including ascertainment of pharmacotherapy in order to characterise further the profiles associated with higher risk.

The terms dictionaries are available to the community at http://git.brc.iop.kcl.ac.uk/rmallah/dystoniaml/. The records themselves are available subject to a collaborative agreement which adheres to strict patient led governance. We would encourage the community to make contact with the authors to establish a collaboration.
